# Housing and Management Practices on 33 Pullet Farms in Canada

**DOI:** 10.3390/ani9020049

**Published:** 2019-02-06

**Authors:** Nienke van Staaveren, Caitlin Decina, Christine F. Baes, Tina M. Widowski, Olaf Berke, Alexandra Harlander-Matauschek

**Affiliations:** 1 Department of Animal Biosciences, Ontario Agricultural College, University of Guelph, Guelph, ON N1G 2W1, Canada; nvanstaa@uoguelph.ca (N.v.S.); cbaes@uoguelph.ca (C.F.B.); twidowsk@uoguelph.ca (T.M.W.); 2 Department of Population Medicine, Ontario Veterinary College, University of Guelph, Guelph, ON N1G 2W1, Canada; cdecina@uoguelph.ca (C.D.); oberke@uoguelph.ca (O.B.)

**Keywords:** aviary, cage, floor system, rearing, chicks, welfare

## Abstract

**Simple Summary:**

The rearing period of pullets plays an important role in the successful transition to furnished cages or non-cage housing systems for laying hens. Changes in the laying hen sector often occur without accompanying changes in the pullet rearing sector, which could pose a risk to the success of the housing system transition. However, little is known about the housing and management practices of pullets worldwide. Recently, a new Code of Practice was released (March 2017) in Canada which provides one of the first guidelines on pullet rearing. This study aimed to describe current housing and management practices applied on pullet farms in Canada, where farmers are in the process of transitioning to this new Code of Practice. Thirty-three pullet farmers were surveyed to understand commonly used management practices in cage (14), single-tier (11) and multi-tier (8) flocks. In general, non-cage farmers met the new requirements set out in the Code of Practice for space, perches and litter provision during pullet rearing. Flocks in conventional cages did not have opportunities for perching and foraging. Further research is needed to develop methods to provide pullets with opportunities to perch and forage in these systems. Additionally, clear litter management recommendations for farmers to ensure good litter quality are needed for non-cage housing systems.

**Abstract:**

Although Canada is one of the first to provide guidelines on pullet rearing in a new Code of Practice which came into effect in March 2017, little information is available about the housing and management of pullets on Canadian farms. We surveyed 99 pullet farmers and received useable responses from 33 pullet farmers (33.3% response rate) who took part in the Start Clean-Stay Clean™ program through their provincial egg boards across Canada during October–December 2017 as part of a larger study. Most flocks were housed in conventional cage systems (42.4%), followed by single-tier (33.3%) and multi-tier systems (24.2%). Flocks ranged from 1–19 weeks of age (average: 10.5 weeks of age) and were white- (58.1%) or brown-feathered (41.9%). In general, non-cage farmers met the new requirements set out in the Code of Practice for space, perches and litter provision during pullet rearing during this transitional period. Conventional caged flocks did not have opportunities for perching and foraging, but developing new methods to provide pullets with opportunities to perch and forage will become more important as the laying hen housing system transition from conventional cages to furnished cage and non-cage housing systems in Canada progresses. Additionally, clear litter management recommendations for farmers to ensure good litter quality are needed for non-cage housing systems.

## 1. Introduction

Early life experiences have long-lasting effects on the brain and body, impacting brain function, behaviour and physical health throughout the life-span of an animal [1]. Domestic chickens are hatched with well-developed brains, and during the first few weeks of life they learn and develop behaviours important for social hierarchy formation, thermoregulation, fear responses, feed searching and spatial navigation [2]. At the same time neuromuscular function and skeletal frame are also developing, contributing to physical fitness, bone health and locomotory abilities [2]. Therefore, the rearing period is a critical phase of life for the overall well-being of the laying hen.

Rearing management can influence bird development and differences in physical and social environments between rearing and laying hen facilities can have large consequences for bird adaptability and productivity in the layer phase [3,4]. Rearing pullets in environments similar to where they will be kept during lay is thought to ease the transition between these phases. This is particularly important for non-cage, and especially aviary systems, when the provision of a generally more complex environment (e.g., perches, foraging- or dustbathing material) helps birds develop skills to navigate and reduces behavioural problems [3,5,6,7,8]. Despite its key role, research into the rearing housing and management of pullets and its effects over a bird’s lifetime has been a relatively neglected field until recently [4]. Additionally, information on the environment and management of pullets is not always known to the laying hen farmer [9], which can be problematic considering the complexity of rearing and egg laying hen housing systems available (ranging from cage systems to multi-tier aviary systems).

European legislation which provides minimum standards for laying hens does not provide recommendations for pullet rearing [10]. Private and organic standards have been increasingly implemented in different countries [11,12], but guidelines for pullet rearing are still limited [13,14]. The Canadian Code of Practice for the Care and Handling of Pullets and Layers provides one of the first guidelines with detailed requirements for space, perches and litter provision during pullet rearing [15]. These requirements were based on available scientific evidence, expert views and industry input through a committee comprised of farmers, animal welfare experts, transporters, processors, veterinarians, and retail and government representatives [16]. That said, there are few recent published reports about the housing and management practices commonly used for pullets worldwide e.g., [17,18,19] and none for Canadian farms.

Recently, van Staaveren et al. [20] surveyed Canadian laying hen farmers and we now present results of a similar survey among 33 pullet farmers throughout Canada. The goal of this study was to describe housing and management practices on pullet farms in light of the upcoming transition from conventional cages to furnished cages and non-cage housing systems in Canada’s laying hen sector [21].

## 2. Materials and Methods

A questionnaire was designed to identify housing, management practices and flock characteristics used on pullet rearing farms similar to that in van Staaveren et al. [20]. In brief, the questionnaire covered topics of housing conditions, litter management, nutrition and feeding, environmental control, flock characteristics, flock rearing and placement, and flock health adapted from [22,23]. Questions were modified to reflect the rearing stage based on the experience of the research team with the Canadian egg production sector. Feedback from the federal and provincial egg boards in Canada and staff from the Arkell Poultry Research Station (Ontario Ministry of Agriculture, Food and Rural Affairs [OMAFRA]—University of Guelph Agreement) as well as local farms was incorporated. This study was approved by the University of Guelph Research Ethics Board (REB17-06-010).

Commercial farmers were invited to participate in the context of a larger cross-sectional study to identify factors associated with feather damage in laying hens [24]. Questionnaires were sent to pullet rearers that were on the Start Clean-Stay Clean™ program through their provincial egg boards across Canada in October 2017. This is a comprehensive food safety and quality program assessed by the Canadian Food Inspection Agency [25]. Farmers were asked to complete the questionnaire for their current flock. Additionally, farmers were provided the opportunity to fill in the questionnaire online via Qualtrics (Qualtrics, Provo, UT, USA). Reminders were sent through provincial egg boards 2–4 weeks after distribution, and two weeks before the end of data collection in December 2017.

Data were entered into Microsoft Office Excel (Professional Plus 2016) using manual double entry. Data were screened for entry errors and invalid responses were considered as missing values. Space allowance was provided by farmers or calculated by the research team based on dimensions of cage or barn/housing section and the number of birds housed within the space as per van Staaveren et al. [20]. Useable vertical space in multi-tier systems could not be calculated, and so values for space allowance should be interpreted with caution. Within the different housing systems, the frequency of different management practices used was determined (SAS v9.3, SAS Inst. Inc., Cary, NC, USA). Results are presented as the number and percentage of flocks for categorical variables and mean ± SD (range: min–max) for continuous variables.

## 3. Results

A total of 99 pullet farmers were invited to participate and 33 useable questionnaires providing housing and management information on pullet flocks were returned (33.3% response rate). Over half of the farmers had more than 10 years of experience in pullet rearing (57.6%). The frequency of housing design, flock characteristics and management reported by farmers, representing data of the 33 pullet flocks in Canada as collected during October–December 2017, is described below. Flocks were placed between May and November 2017 shortly after the new Code of Practice was released in March 2017 and as such data were collected during a transitional period.

### 3.1. Housing

#### 3.1.1. Housing Design

Pullet flocks were sampled from various provinces throughout Canada, mainly Alberta (30.3%), British Columbia (21.2%), Ontario (12.1%), Manitoba (12.1%) and Saskatchewan (9.1%). Fourteen flocks were housed in conventional cage systems (42.4%), while 11 flocks (33.3%) and 8 flocks (24.2%) were housed in single-tier floor systems and multi-tier aviary systems, respectively. Multi-tier systems had 2 (12.5%), 3 (62.5%), or more than 3 tiers (25.0%). Four flocks came from certified organic multi-tier farms. All farms had more than 5000 birds on site and over a third of farms had more than 25,000 birds (Table 1).

#### 3.1.2. Group Size and Space Allowance

Group size was 20 ± 7 birds (range: 11–30) in caged systems, while in single- and multi-tier systems birds were housed in groups of 11,250 ± 7038 birds (range: 5300–27,821) and 11,392 ± 4083 birds (range: 6850–19,910), respectively. Average space allowance in cages was 292.6 ± 75.44 cm^2^/bird (range: 192.6–439.9 cm^2^/bird). For flocks in single-tier systems, this was 875.4 ± 219.53 cm^2^/bird (range: 696.8–1430.9 cm^2^/bird) and in multi-tier systems 715.5 ± 230.42 cm^2^/bird (range: 474.3–1035.8 cm^2^/bird).

#### 3.1.3. Provision of Furnishings and Litter

All flocks in multi-tier systems and nearly all single-tier flocks had access to perches, while no perches were provided for caged pullets (Table 2). In non-cage housing systems, these perches were also provided at different heights (78.5%) and allowed birds to perch at the same time (64.3%). Enrichment was only provided in non-cage housing systems (e.g., bales of hay/straw or mineral blocks/pecking stones) but this was not a common practice (Table 2).

None of the flocks in the cage systems had access to scratch areas which could provide foraging/dustbathing opportunities. On the other hand, litter was provided in all non-cage systems with single-tier systems most often being an all litter barn, while multi-tier systems consisted of a combination of wire, slats and litter (Table 3). In these barns, different proportions of the floor were covered with litter. Sawdust or wood shavings (66.7%) were most commonly used as bedding for litter provision, while others allowed litter to build up over time from manure, feathers and dust in the system (16.7%). Birds received access to the litter at an average of 3.5 weeks of age (range: 1.0–12.0 weeks of age). Litter depth was 9.8 ± 6.87 cm (range: 0.0–25.4 cm) in single-tier and 6.0 ± 5.07 cm (range: 0.0–15.2 cm) in multi-tier systems. Litter was not replaced after first access, and only a few farmers indicated they raked the litter in order to break up the substrate (Table 3).

#### 3.1.4. Nutrition and Feeding

All flocks used nipple drinkers (100%) and the most common feeders were chain (72.7%) or pan feeders (18.2%). Feed was often given as mash and was equally bought from external sources or home-milled (Table 4). Changes in the diet occurred as birds became older and were often gradually introduced (62.1%). Typically, birds had changed diets 0–2 times within 7 weeks of age while >3 changes had occurred by the time birds reached approximately 12 weeks. Feeders were operated an average of 4.3 ± 1.68 times per day (range 2–10 times). Practices such as midnight feeding (3.0%) or providing animal by-products in the diet (25.0%) only occurred in a small proportion of flocks. Nearly two-thirds of the flocks (62.1%) were provided with supplements such as insoluble grit (6.9%), insoluble fibre (24.1%), oyster shell (13.8%) and vitamins (44.8%).

#### 3.1.5. Environmental Control

Pullets received on average 10.9 ± 2.09 h of light per day (range: 9.0–18.0 h), mostly from LED sources. Light intensity as measured at bird height on the lowest level was between 5 to 15 lux on most farms (66.7%), though not all farmers were able to answer this question (18%). Two-thirds of farmers provided a dawn/dusk period (66.7%), and this was most common on farms with multi-tier systems. Dawn/dusk was simulated by the gradual dimming of lights in different areas in multi-tier systems, while all lights were dimmed automatically in cages and single-tier systems.

Barn climate was controlled through mechanical ventilation on most farms (90.9%). Average temperature and humidity were 22.1 ± 2.83 °C (range: 19.0–34.0 °C) and 54.4 ± 14.98% relative humidity (RH; range: 22.0–75.0% RH), respectively. The removal of manure occurred at least once per week in cage or multi-tier systems via manure belt, while in single-tier systems manure was removed at the end of the flock on most farms (Table 5).

### 3.2. Flock Characteristics

#### 3.2.1. General Characteristics

Pullet flocks were 10.5 ± 5.22 weeks old (range: 1–19 weeks) at the time of the study. All flocks were beak trimmed (approx. ¼ of the length of the beak) at day 1 in the hatchery using infrared beak treatment. Flocks were either white- (58.1%) or brown-feathered (41.9%) and mostly of the Lohmann, Dekalb, H&N and Hy-line breed (Table 6). In cages, most flocks were white-feathered, while in single-tier systems most were brown-feathered, and an equal number of white- and brown-feathered flocks were housed in multi-tier systems.

#### 3.2.2. Flock Rearing and Transfer to Laying Farms

Most flocks were to be transferred to laying facilities on the same farm and never mixed during rear (Table 7). Farmers discussed the rearing conditions in the barn with the laying farmers (87.1%), and over 90% indicated they attempted to match conditions between rearing and laying facilities (Table 7).

#### 3.2.3. Flock Health

Flock inspections occurred 2–3 times per day (78.8%) and were performed by 1–2 workers (97%) (Table 8). Nearly all farmers reported to always inspect the bottom and top tier (97%) and 69% indicated they sometimes handled birds during inspection. Daily inspections took relatively more time in multi-tier and cage systems compared to single-tier systems. Route taken during inspection varied according to approximately two-thirds of the farmers. Health (100%) and behaviour (90.9%) of the birds, functioning of equipment (93.9%) and litter quality (78.8%) were the main aspects checked during inspection. Barns were disinfected before arrival of the birds (100%) and dedicated clothing and/or clean boot dips were used (97%). A smaller proportion of farmers (43.8%) had developed and implemented a flock health plan with their veterinarian. All flocks were vaccinated though there were differences between the flocks against which diseases they were vaccinated (Table 8). The main diseases flocks were vaccinated against included Marek’s disease, infectious bursal disease, Newcastle disease, infectious bronchitis, avian encephalomyelitis, *E. coli*, and coccidiosis, while only a few flocks vaccinated against fowl pox, infectious laryngotracheitis and salmonella. No diseases had been observed since placement in the majority of flocks (87.1%). Average cumulative mortality was 1.8 ± 1.67% (range 0.005–8.33%), and reasons for mortality included birds getting trapped in housing equipment (28.2%), leg injuries (24.4%), disease (21.8%), and smothering (15.4%).

## 4. Discussion

### 4.1. Housing

This study is the first to present results on housing and management practices applied on 33 pullet farms in Canada and addresses the urgent need for up-to-date information on these aspects of pullet rearing worldwide e.g., [17,18,19]. It should be kept in mind that this sample was recruited through the provincial egg boards and pullet farmers who were on their Start Clean-Stay Clean™ program under the Egg Farmers of Canada were included. This is a comprehensive food safety and quality program assessed by the Canadian Food Inspection Agency [25]. Additionally, as this study was part of a larger project aimed at identifying management factors associated with feather damage in laying hens kept in furnished cage and non-cage housing systems [24], nearly all pullet farmers provided birds for their own laying farms and not all pullet farmers on the Start Clean-Stay Clean™ program were contacted to participate in the study. Therefore, these findings should not be generalized to the pullet sector as a whole in Canada. Surveyed pullet farmers represented the major egg production provinces across Canada and it should be noted that large differences in climate across the country [26] could have implications for management practices. Flocks were placed after the National Farm Animal Care Council published the new Code of Practice that came into effect in March 2017, which provides some of the first guidelines for pullet housing and management [15]. While this new Code of Practice should be followed in management of these flocks it is important to be aware of the short timeframe between release of the Code of Practice and the survey in the current study and that a transitional period is needed to make structural modifications.

The majority of study flocks were housed in conventional cage systems, while around a third were housed in single-tier rearing systems and nearly a quarter of the flocks were reared in multi-tier rearing systems. This is not surprising, as there are currently few furnished cages for rearing pullets available on the market, and multi-tier pullet rearing systems are only recently being used in Canada with many now on their first few flocks. With the upcoming transition to furnished cage and non-cage housing systems within the Canadian egg production sector [21], it should be noted that there do not appear to be accompanying changes within the pullet sector in terms of housing system changes or financial compensation. However, it is likely that these systems will gain more use as changes in the laying hen sector trickle down to the pullet sector.

On average, farmers meet the requirements for space allowance of pullets in cages, single-tier and multi-tier systems [15], with the range in space allowance observed being explained by the large range of bird ages. Space allowance should increase as birds get older, but few recommendations have been made previously for pullet space allowance [3]. The new Code of Practice requires that pullets of 8 weeks of age should receive 283.9 cm^2^/bird in cages and multi-tier systems (plus litter space 58.1 cm^2^/bird in multi-tier systems), and 696.8 cm^2^/bird in single-tier systems [15].

Physical, sensory and stimulatory additions to the environment of the birds can help maximize the birds’ developmental potential [27]. The importance of providing perching and foraging opportunities early in life have been well documented [3,28,29]. As such, caged pullet flocks are not allowed to transition to non-cage housing systems under the Code of Practice [15]. Currently, perching and foraging opportunities are not available in pullet rearing cage systems, which limits the ability to match rearing to layer housing for hens destined for housing in furnished cages during the laying period [4]. However, the vast majority of non-cage flocks surveyed in this study provided perches and all provided pullets with access to litter. Only a few of the flocks housed in single-tier systems did not have access to perches, which will likely be addressed following a transitional period while farmers make these structural changes to their housing system. Providing perches is a recommended practice for pullets destined for single-tier housing and required practice for pullets destined for multi-tier housing in the new Code of Practice [15]. Huber-Eicher [17] reported that approx. half of 66 surveyed pullet farms in Switzerland (single-tier and multi-tier systems) provided non-elevated perches, while 6% did not provide perches at all. More recent data are not available. In our study, access to litter was provided when pullets were on average 3.5 weeks of age. Final requirements in the Code of Practice state that about 17% of the space allowance available per bird should be litter space (58.1 cm^2^ in addition to 283.9 cm^2^ per pullet at 8 weeks of age or older) [15]. While specific litter space allowance per bird could not be determined, we asked farmers to estimate the proportion of barn surface that was covered with litter. The majority of single-tier systems had all litter barns, while in multi-tier systems a varied proportion of the barn surface was covered with litter. A total of 75 percent of multi-tier systems had ≥1/3 of the barn floor covered with litter. In comparison, previous surveys on rearing flocks in Switzerland conducted over 20 years ago found that more than 70% of floor space was littered in single-tier systems (670 cm^2^ per bird), while in aviary systems 326 cm^2^ per bird in litter space was available [17]. While there are no clear recommendations on litter space provision, it is important to consider the quality and maintenance of the litter substrate which is a buildup of bedding material and manure, feathers and dust in the systems. Often bedding is provided in the form of sawdust and shavings, while around 16% of farmers indicated that they let manure, feathers and dust build up in the system. Manure was more frequently removed in cage and multi-tier systems due to the structural differences, with these systems allowing farmers the use of manure belts. However, manure in the single-tier and the littered area of multi-tier systems builds up which can have implications for ammonia control, enhance litter deterioration and be aversive to the birds [30,31,32]. Finally, litter is typically not replaced or broken up over the rearing period which could affect the quality of the litter substrate. However this is also dependent on other factors such as barn climate and water spillage which play a role in litter friability [33,34,35]. If litter is no longer loose or friable, birds do not use it for its intended purpose of foraging or dustbathing [32].

Feed was mostly provided in mashed form, though crumbs were also given similarly as reported by Huber-Eicher [17] depending on the age of the flock. Diet changes were more frequently reported in the current study than in the Swiss study [17], however, diet changes reported in the current study were in line with breed guidelines [36,37]. The number of times feeders ran per day was similar to reported frequencies of 5.5 times per day [17]. Environmental control was largely dependent on the different ages of the flocks and was in line with common breed guidelines for the duration of light, temperature and humidity levels [36,37]. Both diet changes and environmental factors have previously been implicated in the development of feather pecking at rear and at lay [38].

### 4.2. Flock Characteristics

Flocks in the study covered a large range of ages (1–19 weeks of age) with an average age of 10 weeks. Pullet development occurs rapidly within the first weeks of life and can explain the variation in management practices observed in this study. There was an equal representation of white- and brown-feathered flocks with the most common breeds being Dekalb White, H&N Nick Chick, Lohmann Brown and Lohmann LSL. Most pullet flocks were being reared for egg production in the farmers own laying facilities, however, a proportion of flocks was intended for egg production on external laying farms. The high proportion of farmers raising pullets for their own laying hen flocks is likely a consequence of the recruitment of pullet farmers on the Start Clean-Stay Clean™ program by the provincial egg boards.

In general, the surveyed flocks showed little disease and relatively low mortality rates. This can be attributed to the hygiene measures (e.g., disinfection of barns, dedicated clothing and/or clean boot dips, frequent inspections of flocks) and a rigorous vaccination scheme [39]. There is some variation amongst farmers with respect to the type of diseases they vaccinated their flocks against, as also seen in other organic pullet farmers in the UK [19]. Mostly, this variation is observed in those vaccinations which are considered optional in breed guidelines and are only recommended when the disease is present in the area [40]. Veterinarians were included in the development and implementation of flock health plans in around 44% of the flocks which is similarly reported by others [19]. This involvement seems higher compared to that in laying hen flocks [20]. However, similar to laying hen flocks, visits by veterinarians to farms were limited [19,20]. While disease was mentioned as a reason for mortality, other reasons not related to disease were more frequently reported. This highlights the importance of adequate housing design and good human-animal relationships to avoid issues such as pullets getting trapped in housing equipment, leg injuries and smothering [3].

## 5. Conclusions

Housing and management practices applied on 33 Canadian pullet rearing farms are reported here for the first time. This study addresses the limited information available on commercial pullet rearing and shows an uptake of requirements regarding space, perches and litter provision during pullet rearing set out in the new Code of Practice, with the exception of perching and foraging opportunities in conventional caged flocks. Development of new methods to provide pullets with opportunities to perch and forage in these systems will become more important as the laying hen housing system transition from conventional cages to furnished cage and non-cage housing systems in Canada progresses. Additionally, clear litter management recommendations for farmers to ensure good litter quality are needed for non-cage housing systems.

## Figures and Tables

**Table 1:** A description of general farm characteristics for 33 pullet flocks placed between May and November 2017 according to housing system (cage *n* = 14, single-tier *n* = 11, multi-tier *n* = 8) in Canada.

Variable	Cage		Single-Tier		Multi-Tier	
	*n*	%	*n*	%	*n*	%
No. of hens						
Less than 1000	0	0.0	0	0.0	0	0.0
1000–5000	0	0.0	0	0.0	0	0.0
5000–10,000	5	35.7	6	54.6	2	25.0
10,000–15,000	1	7.1	1	9.1	2	25.0
15,000–20,000	0	0.0	2	18.2	1	12.5
20,000–25,000	1	7.1	0	0.0	0	0.0
More than 25,000	7	50.0	2	18.2	3	37.5
Age of system						
Less than 1 yr	0	0.0	0	0.0	0	0.0
1–4 years	2	14.3	6	66.7	5	71.4
5–10 years	2	14.3	1	11.1	1	14.3
More than 10 years	10	71.4	2	22.2	1	14.3

**Table 2:** Distribution of perches and enrichment provision for 33 pullet flocks placed between May and November 2017 according to housing system (cage *n* = 14, single-tier *n* = 11, multi-tier *n* = 8) in Canada.

Variable	Cage		Single-Tier		Multi-Tier	
	*n*	%	*n*	%	*n*	%
Perches						
Yes	0	0.0	9	81.8	8	100.0
No	14	100.0	2	18.2	0	0.0
Perches at different heights ^1^						
Yes	NA	NA	6	75.0	8	100.0
No	NA	NA	2	25.0	0	0.0
Space to perch at the same time ^1^						
Yes	NA	NA	6	66.7	3	60.0
No	NA	NA	3	33.3	2	40.0
Enrichment provided						
Yes	0	0.0	2	20.0	1	12.5
No	14	100.0	8	80.0	7	87.5

^1^ Only answered by farmers who indicated that they provided perches.

**Table 3:** Summary of litter management aspects for pullet flocks placed between May and November 2017 according to housing system (single-tier *n* = 11, multi-tier *n* = 8) in Canada.

Variable	Single-Tier		Multi-Tier	
	*n*	%	*n*	%
Type of barn				
All litter barn	10	90.9	0	0.0
Combination of wire, slats and litter	1	9.1	8	100.0
All wire/slatted barn	0	0.0	0	0.0
Proportion of barn with litter ^1^				
<1/3 of barn	1	100.0	2	25.0
1/3 of barn	0	0.0	3	37.5
>1/3 of barn	0	0.0	3	37.5
Type of litter				
Sand	0	0.0	0	0.0
Wood shavings/sawdust	7	63.6	5	71.4
Straw	2	18.2	0	0.0
Combination of the above	1	9.1	0	0.0
None/manure	1	9.1	2	28.6
Litter replaced after first access				
Yes	0	0.0	0	0.0
No	11	100.0	8	100.0
Frequency of breaking up of litter				
Never	10	90.9	6	75.0
Seasonally	0	0.0	0	0.0
Monthly	1	9.1	2	25.0
Weekly	0	0.0	0	0.0
Daily	0	0.0	0	0.0

^1^ Only answered by farmers who indicated that they had a barn with ‘combination of wire, slats and litter’.

**Table 4:** Frequency of nutrition and feeding practices for 33 pullet flocks placed between May and November 2017 according to housing system (cage *n* = 14, single-tier *n* = 11, multi-tier *n* = 8) in Canada.

Variable	Cage		Single-Tier		Multi-Tier	
	*n*	%	*n*	%	*n*	%
Feed structure						
Mashed feed	8	57.1	11	100.0	6	75.0
Pelleted feed	2	14.3	0	0.0	1	12.5
Grains	0	0.0	0	0.0	1	12.5
Crumbs	4	28.6	0	0.0	0	0.0
Feed source						
Home-milled	6	42.9	6	54.6	3	37.5
Purchased	8	57.1	5	45.5	3	37.5
Combination	0	0.0	0	0.0	2	25.0
No. of times diet has been changed						
No changes	2	14.3	2	18.2	0	0.0
1×	1	7.1	2	18.2	1	12.5
2×	0	0.0	2	18.2	2	25.0
3×	4	28.6	2	18.2	0	0.0
4×	5	35.7	3	27.3	2	25.0
More than 4×	2	14.3	0	0.0	3	37.5
Method of changing diet ^1^						
Gradual change	5	41.7	7	77.8	6	75.0
Immediate change	7	58.3	2	22.2	2	25.0
Midnight feeding						
Yes	1	7.1	0	0.0	0	0.0
No	13	92.9	11	100.0	8	100.0
Supplements provided						
No	6	50.0	3	27.3	2	33.3
Yes	6	50.0	8	72.7	4	66.7
Insoluble grit ^2^	0	0.0	1	9.1	1	16.7
Insoluble fibre ^2^	3	25.0	2	18.2	2	33.3
Oyster shell ^2^	2	16.7	2	18.2	0	0.0
Vitamins ^2^	3	25.0	7	63.6	3	50.0
Animal by-products in feed						
Yes	5	38.5	0	0.0	3	37.5
No	8	61.5	11	100.0	5	62.5

^1^ Only answered by farmers who indicated that they had changed the diet of the flock. ^2^ Those farmers who indicated that they provided supplements were asked to indicate if they provided: insoluble grit, insoluble fiber, oyster shell, and/or vitamins.

**Table 5:** Frequency of environmental control practices for 33 pullet flocks placed between May and November 2017 according to housing system (cage *n* = 14, single-tier *n* = 11, multi-tier *n* = 8) in Canada.

Variable	Cage		Single-Tier		Multi-Tier	
	*n*	%	*n*	%	*n*	%
Type of lighting						
Incandescent	4	28.6	4	36.4	1	12.5
Fluorescent	4	28.6	1	9.1	0	0.0
LED	6	42.9	6	54.6	7	87.5
Dawn/dusk period provided						
Yes	8	57.1	6	54.6	8	100.0
No	6	42.9	5	45.5	0	0.0
Method of dawn/dusk provision ^1^						
All lights dimmed	7	87.5	5	83.3	2	25.0
Gradual dimming by area	1	12.5	1	16.7	6	75.0
Light intensity						
Less than 5 lux	3	27.3	2	20.0	2	33.3
5–10 lux	3	27.3	6	60.0	1	16.7
11–15 lux	4	36.4	1	10.0	3	50.0
16–20 lux	0	0.0	0	0.0	0	0.0
21–25 lux	0	0.0	0	0.0	0	0.0
More than 25 lux	1	9.1	1	10.0	0	0.0
Type of ventilation						
Controlled (fan)	14	100.0	8	72.7	8	100.0
All natural	0	0.0	0	0.0	0	0.0
Combination (fan + natural)	0	0.0	3	27.3	0	0.0
Manure removal						
1× per day	2	14.3	0	0.0	0	0.0
3× per week	3	21.4	0	0.0	0	0.0
2× per week	4	28.6	1	10.0	4	50.0
1× per week	4	28.6	0	0.0	1	12.5
Every 3 weeks	1	7.1	0	0.0	0	0.0
End of flock	0	0.0	9	90.0	3	37.5

^1^ Only answered by farmers who indicated that they provided a dawn/dusk period.

**Table 6:** Frequency of flock characteristics for 33 pullet flocks placed between May and November 2017 according to housing system (cage *n* = 14, single-tier *n* = 11, multi-tier *n* = 8) in Canada.

Variable	Cage		Single-Tier		Multi-Tier	
	*n*	%	*n*	%	*n*	%
Feather colour						
Brown	2	16.7	7	63.6	4	50.0
White	10	83.3	4	36.4	4	50.0
Breed						
Dekalb White	1	9.1	0	0.0	2	33.3
Hisex Brown	0	0.0	1	9.1	0	0.0
H&N Nick Chick	2	18.2	1	9.1	0	0.0
Hy-line Brown	1	9.1	1	9.1	0	0.0
ISA Brown	0	0.0	1	9.1	0	0.0
Lohmann Brown Classic	0	0.0	1	9.1	1	16.7
Lohmann Brown Lite	1	9.1	2	18.2	2	33.3
Lohmann LSL Lite	5	45.5	3	27.3	1	16.7
Novogen	0	0.0	1	9.1	0	0.0
Shaver	1	9.1	0	0.0	0	0.0

**Table 7:** Description of flock rearing and placement practices for 33 pullet flocks placed between May and November 2017 according to housing system (cage *n* = 14, single-tier *n* = 11, multi-tier *n* = 8) in Canada.

Variable	Cage		Single-Tier		Multi-Tier	
	*n*	%	*n*	%	*n*	%
Supply farm						
Own laying farm	11	78.6	6	54.6	4	50.0
External laying farm(s)	1	7.1	0	0.0	1	12.5
Combination	2	14.3	5	45.5	3	37.5
Discuss rearing with laying farm						
Yes	13	92.9	9	81.8	5	83.3
No	1	7.1	2	18.2	1	16.7
Match conditions barn to laying						
No	2	14.3	1	9.1	0	0.0
Yes	12	85.7	10	90.9	8	100.0
Housing ^1^	8	66.7	8	80.0	6	75.0
Perches ^1^	0	0.0	6	60.0	4	50.0
Litter ^1^	2	16.7	5	50.0	5	62.5
Feed ^1^	12	100.0	9	90.0	7	87.5
Environment ^1^	12	100.0	9	90.0	7	87.5
Slats ^1^	0	0.0	2	20.0	0	0.0

^1^ Those farmers who indicated that they matched conditions between rearing and laying facilities were asked whether they do this for the following aspects: housing, perches, litter, feed and environment.

**Table 8:** Frequency of flock health management practices for 33 pullet flocks placed between May and November 2017 according to housing system (cage *n* = 14, single-tier *n* = 11, multi-tier *n* = 8) in Canada.

Variable	Cage		Single-Tier		Multi-Tier	
	*n*	%	*n*	%	*n*	%
No. of bird inspections per day						
1×	2	14.3	1	9.1	1	12.5
2×	8	57.1	6	54.6	4	50.0
3×	2	14.3	3	27.3	3	37.5
4×	0	0.0	0	0.0	0	0.0
5×	0	0.0	0	0.0	0	0.0
More than 5×	2	14.3	1	9.1	0	0.0
No. of workers						
1 worker	9	64.3	6	54.6	6	75.0
2 workers	4	28.6	5	45.5	2	25.0
3 workers	1	7.1	0	0.0	0	0.0
More than 3 workers	0	0.0	0	0.0	0	0.0
Average time spent on inspection per day						
Less than 15 min	1	7.1	1	9.1	0	0.0
15–30 min	4	28.6	3	27.3	3	37.5
30–45 min	2	14.3	5	45.5	1	12.5
45–60 min	4	28.6	2	18.2	4	50.0
More than 60 min	3	21.4	0	0.0	0	0.0
Vary route during inspections						
Yes	8	57.1	7	70.0	6	75.0
No	6	42.9	3	30.0	2	25.0
Flock health plan implemented						
Yes	7	53.9	3	27.3	4	50.0
No	6	46.2	8	72.7	4	50.0
Vaccination of birds						
Marek’s disease	14	100.0	9	90.0	8	100.0
Newcastle disease	14	100.0	10	100.0	8	100.0
Infectious bronchitis	13	92.9	10	100.0	8	100.0
Avian encephalomyelitis	12	85.7	7	77.8	7	87.5
Infectious bursal disease	11	78.6	8	80.0	6	75.0
E. Coli	4	28.6	7	70.0	8	100.0
Coccidiosis	4	28.6	4	40.0	5	62.5
Salmonella	4	28.6	2	20.0	3	37.5
Fowl pox	2	14.3	0	0.0	3	37.5
Infectious laryngotracheitis	2	14.3	2	20.0	2	25.0
Mycoplasmosis	0	0.0	0	0.0	0	0.0
Infectious coryza	0	0.0	0	0.0	0	0.0
Fowl cholera (pasteurellosis)	0	0.0	0	0.0	0	0.0
Egg drop syndrome	0	0.0	0	0.0	0	0.0
Avian pneumovirus	0	0.0	0	0.0	0	0.0
Diseases observed since placement						
Yes	2	14.3	1	11.1	1	12.5
No	12	85.7	8	88.9	7	87.5
